# Winter Active Bumblebees (*Bombus terrestris*) Achieve High Foraging Rates in Urban Britain

**DOI:** 10.1371/journal.pone.0009559

**Published:** 2010-03-05

**Authors:** Ralph J. Stelzer, Lars Chittka, Marc Carlton, Thomas C. Ings

**Affiliations:** 1 School of Biological and Chemical Sciences, Queen Mary University of London, London, United Kingdom; 2 The London Natural History Society, London, United Kingdom; Trinity College Dublin, Ireland

## Abstract

**Background:**

Foraging bumblebees are normally associated with spring and summer in northern Europe. However, there have been sightings of the bumblebee *Bombus terrestris* during the warmer winters in recent years in southern England. But what floral resources are they relying upon during winter and how much winter forage can they collect?

**Methodology/Principal Findings:**

To test if urban areas in the UK provide a rich foraging niche for bees we set up colonies of *B. terrestris* in the field during two late winter periods (2005/6 & 2006/7) in London, UK, and measured their foraging performance. Fully automatic radio-frequency identification (RFID) technology was used in 2006/7 to enable us to record the complete foraging activity of individually tagged bees. The number of bumblebees present during winter (October 2007 to March 2008) and the main plants they visited were also recorded during transect walks. Queens and workers were observed throughout the winter, suggesting a second generation of bee colonies active during the winter months. Mass flowering shrubs such as *Mahonia* spp. were identified as important food resources. The foraging experiments showed that bees active during the winter can attain nectar and pollen foraging rates that match, and even surpass, those recorded during summer.

**Conclusions/Significance:**

*B. terrestris* in the UK are now able to utilise a rich winter foraging resource in urban parks and gardens that might at present still be under-exploited, opening up the possibility of further changes in pollinator phenology.

## Introduction

**Figure 1:**
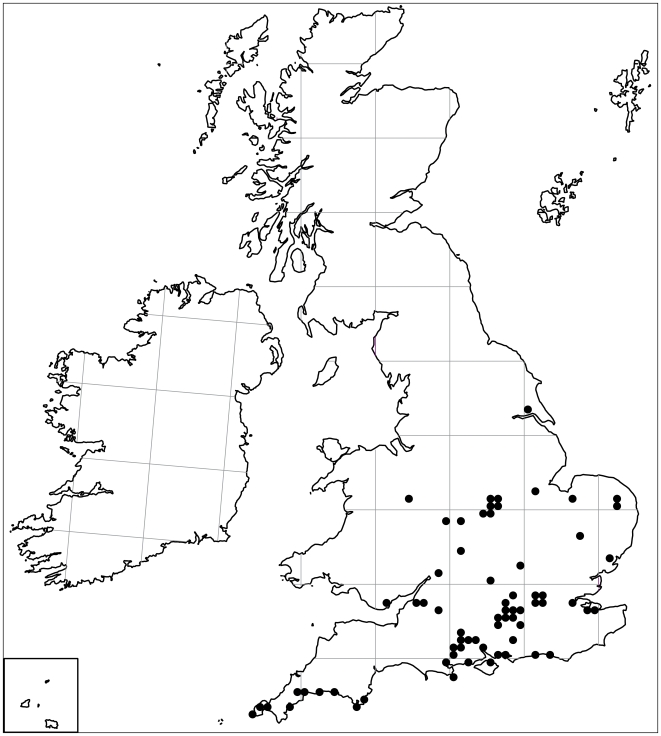
Distribution of winter active *B. terrestris* in the UK from October 2008 to March 2009. Data (247 records of workers and 329 of queens) were kindly provided by the Bees, Wasps and Ants Recording Society and the map was produced by Stuart Roberts.

Bumblebees in northern Europe typically have one, or in a few species, two generations that are active during the spring and summer [1], [2]. Colonies perish in the autumn and only newly mated queens survive the winter by hibernating. However, in recent years, foraging bumblebees, *Bombus terrestris* (L.), have been repeatedly observed during winters in southern England [1], [3], [4]. The first report in the literature dates back to 1990, when several *B. terrestris* workers and queens were seen in Exeter, UK [5]. In the following years the number of ancedotal reports about winter foraging bumblebees increased, especially in London and the surrounding Home Counties, but also from more northern areas such as Shropshire and even south-east Yorkshire (Kingston upon Hull) (Fig. 1).

Although queens of *B. terrestris* emerging from hibernation during midwinter are not uncommon after warm spells, even in otherwise colder winters [6], recent observations of bees in the winter include workers as well as males and nest-founding (collecting pollen) queens [1], [7]. These *ad hoc* sightings therefore suggest that *B. terrestris* may be establishing a second generation during the autumn/winter in southern Britain. Whilst populations of *B. terrestris* living at lower latitudes, i.e. experiencing milder winters (e.g. Mediterranean regions or New Zealand), are known to be able to found an autumn/winter generation [8]–[12], this is unprecedented in the UK, where winter diapause was believed to be obligatory [2], [13].

**Figure 2:**
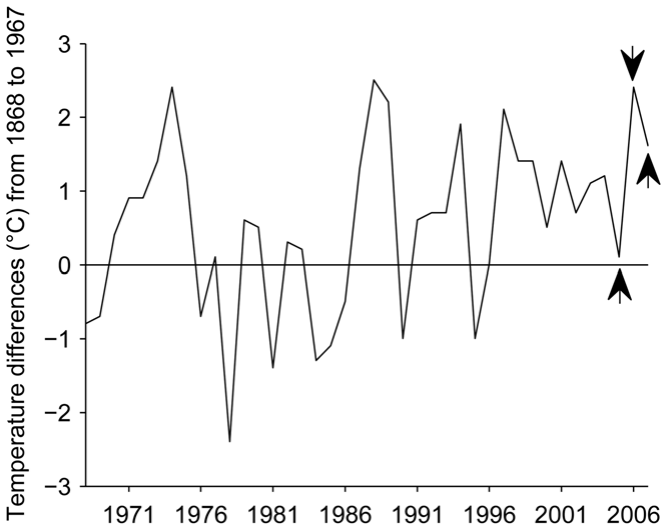
Central England temperature anomalies for the winters of 1968/69 to 2007/08. The graph shows annual anomalies for the mean winter temperature (December to February) of the last 40 years before our observations relative to the average winter temperatures of the preceding 100 (1869/69 to 1967/68) according to the Hadley Centre Central England Temperature (HadCET) database (see [14]). The arrows indicate our foraging rate observations in the winters of 2005/06 (colony A) and 2006/07 (colonies B and C) and our bee survey in Kew Gardens (2007/08). Years on the x-axis indicate December of the according winter, i.e. 1971 stands for winter of 1971/72 and so on.

It is tempting to speculate that the major shift in phenology of *B. terrestris* in the UK is linked to warmer winters in recent years (Fig. 2; see also [14]), possibly aggravated in large cities (urban heat islands) where temperatures are generally higher [15]. However, a first important step towards understanding this new phenomenon is to determine whether winter active bees are able to obtain sufficient resources to sustain a winter population. Although bumblebees are able to forage at ambient temperatures close to 0°C [16], they must find enough nectar and pollen during winter to sustain their colony. In Mediterranean regions, autumn/winter active *B. terrestris* rely heavily upon naturally abundant winter flowering plants such as *Arbutus unedo*, which can provide extensive nectar and pollen rewards [10], [11]. However, in the UK, most native flowers are not in bloom during winter and bumblebees must obtain nectar and pollen from introduced winter flowering plants, which are frequently planted in gardens and parks in urban areas. Are these resources providing a rich foraging niche for winter active bumblebees? Foraging performance is a good measure of bumblebee colony fitness because reproductive success is linked to food supply [17], [18]. Therefore, to test the hypothesis that urban parks and gardens provide a rich winter foraging niche, we monitored activity of *B. terrestris* in London, UK, and recorded the foraging performance of experimental colonies during two winters.

## Materials and Methods

### Experimental Colonies for Nectar and Pollen Foraging Observations

To assess the nectar and pollen foraging rates that can be achieved by *B. terrestris* during late winter, we collected two sets of data on nectar foraging (during the winters of 2005/2006 and 2006/2007) and one set on pollen foraging in the winter of 2006/2007. Since *Bombus terrestris audax* (Harris), the native population in the UK, is not commercially available we used *B. t. dalmatinus* (Dalla Torre), a population originally native in south-eastern Europe, for our observations. For this study, three colonies (A, B and C) were purchased from a commercial breeder (Koppert Biological Systems, Berkel en Rodenrijs, The Netherlands) shortly before they were set up in the field. Colonies A and B were used for measuring nectar foraging rates (A in 2005/2006, B in 2006/2007), colony C for pollen foraging observations (2006/2007). The colonies were housed in bipartite plywood nest boxes (28×16×11 cm) covered with Plexiglas lids and were set up in a glasshouse on the roof of the Fogg Building of the School of Biological and Chemical Sciences, Queen Mary University of London, UK (0.0° W, 51.5° N). The entrance of these boxes consisted of a long transparent tunnel with a system of shutters to enable movements of bees into and out of the nest to be controlled by the observer. Since *B. t. dalmatinus* is not native in England, the colonies were checked daily for males and new unfertilised queens (gynes), which were immediately removed from the colony to prevent accidental establishment of this subspecies in the UK. Before the colonies were introduced to the field, they were fed *ad libitum* with pollen and artificial nectar supplied with the colonies.

### Nectar Foraging Observations

Colony A was set up in the glasshouse between 10/02/2006 and 15/03/2006. Nectar foraging observations began on 17/02/2006 and continued until 15/03/2006. To maximise the number of foraging trips measured for each bee, the measurements were taken over several days during periods of greatest foraging activity (mainly during dry days above 3°C). Nectar foraging rates of 10 individually marked bees were measured. During the observations, all bees were allowed to leave and enter the nest at will. The mass of all marked workers was measured on each departure from and arrival to the nest to calculate the amount of nectar collected ( =  net change in body mass). Departing workers entered a black film canister (of known weight) through a trap door in the entrance tunnel. The canisters were weighed on an electronic balance (Ohaus Navigator N20330, Ohaus Corporation, Pine Brook, NJ, USA) and the workers were released at the tunnel entrance. Returning foragers were captured and weighed in the same way before being reintroduced into the nest box.

Colony B was set up in the glasshouse between 26/01/2007 and 07/02/2007. Nectar foraging observations were performed from 31/01/2007 until 06/02/2007 (excluding 03/02/2007) from about 1100 h until sunset at about 1645 h. Here we fully automated the measurements of foraging performance, since a new technology, radio-frequency identification (RFID) had become available to record the complete foraging activity of individually tagged workers during the course of the experiments [19]–[21]. Small RFID tags (mic3®-TAG 64 bit RO, iID2000, 13.56 MHz system, 1.0×1.6×0.5 mm; Microsensys GmbH, Erfurt, Germany) were glued to the dorsal surface of the thorax of 64 foragers. An RFID reader (iID2000, 2k6 HEAD; Microsensys GmbH, Erfurt, Germany) was integrated into the Plexiglas tunnel close to the nest entrance. All bees were allowed to leave and enter the nest at will during this period. The RFID reader automatically recorded the identity of passing tagged foragers, date and time, as well as the direction of movement of the bee (in or out of the hive entrance) over the whole duration of the experiment.

To automatically measure the body mass of exiting and returning foragers an electronic balance (see above) was integrated into the Plexiglas tunnel between the nest exit and the RFID reader during this experiment. The bees had to enter a small, elongated plastic box lying on the balance: a small clearance gap between the ends of the box and the Plexiglas tube ensured that only the masses of bees inside the box were measured. Bees spent sufficiently long traversing the box to enable stable weight measurement. As the movements of bees were not restricted in any way the weighing box was fitted with a Plexiglas window to determine if more than one bee was in the box at the same time (such readings were discarded). During the observations, a video camera recorded the display of the balance, the RFID reader and a stopwatch, which was synchronised to the reader time. Later, the data from the RFID reader were associated with the corresponding measured weights recorded on videotape.

To calculate the nectar foraging rates achieved by both colonies the following data were recorded: (1) BeeID, (2) departure time, (3) departure mass, (4) arrival time, (5) arrival mass and (6) it was noted if the bee carried pollen or not. For colony A the departure time was taken when the bee was released after weighing, the arrival time was taken when the bee arrived at the entrance of the tunnel system. For colony B departure and arrival time were taken as recorded by the RFID reader when exiting or returning bees passed it.

### Pollen Foraging Observations

Pollen loads of returning foragers were measured using colony C (19/02/07 to 29/03/07). A total of 98 workers were marked with RFID tags in this colony. Again, all bees were allowed to enter and leave the nest at will during this period. Pollen foraging observations took place on 4 days (07/03/07 – 09/03/07 and on 12/03/07) at different times between 1100 h and 1600 h for a maximum of 2 hours on each day to avoid stressing the foragers. The exact time at which returning tagged foragers with pollen loads passed the RFID reader was noted.

After passing the reader, the bees were trapped in a special segment of the tunnel using the shutter system. One side of this tunnel segment consisted of a metal mesh. A soft plunger was located at the other side. By pushing the plunger, the bees were gently pressed against the mesh to retain them. One randomly chosen pollen load was then carefully removed through the mesh using a cocktail stick and weighed immediately using an electronic balance (see above). The bees were afterwards allowed to enter the nest. Only one pollen load was removed to minimise the chances of workers losing their motivation to forage [22]. After weighing, the pollen was deposited into the nest chamber of the nest box. During the weighing process other bees returning with pollen loads were allowed to enter the nest at will. Later, the RFID data (departure and arrival times) were matched to the corresponding pollen load to calculate foraging rates. To calculate the pollen foraging rates the following data were used: (1) BeeID, (2) departure time, (3) arrival time and (4) mass of the pollen load.

### Data Analysis

All flights of less than 5 minutes (or flights above 5 min where bees lost mass) during the nectar and pollen foraging observations were discarded, since the great majority of these flights seem to have been orientation or defecation flights [23], [24]. This happened only rarely (13 excluded flights for colony A, 8 flights for colony B). During the nectar foraging observations, all foragers that returned with pollen were excluded from the analysis. Foraging bouts for which it was not possible to get a stable measurement for the arrival body mass of a bee on the balance integrated in the tubing system were also excluded from the analysis. For foraging bouts where a stable measurement of the body mass of the departing bee was not obtained we used the mean departure body mass of that bee from all of its other foraging bouts. The following foraging parameters were calculated for each bee: (1) mass of nectar or pollen collected, (2) bout duration and ultimately (3) the nectar foraging rate (NFR, mg nectar h^−1^) or pollen foraging rate (PFR, mg pollen h^−1^). Bees with fewer than three foraging bouts were not included in the analysis. The means of the remaining values were calculated for each bee and these means were used as the unit of replication. To calculate the total pollen load collected during a foraging bout, the mass of the one measured pollen load during that bout was doubled (based upon the assumption that both pollen loads weigh the same).

### Transect Walks

To determine whether urban parks and gardens are able to support bumblebees throughout the winter we monitored foraging bees along a fixed transect walk in the Royal Botanic Gardens, Kew, Surrey, UK (hereafter Kew Gardens) on 27 occasions from 01/10/2007 to 28/03/2008. Each walk followed a standard route (∼3 km, including a 300 m double-back section) lasting approximately 40–50 minutes. Along the transect route there were approximately 14 (depending upon date and flowering phenology) flower beds containing winter and early spring flowering plants. Upon arrival at each flower bed the number of *B. terrestris* individuals observed foraging was noted, as well as the plant species they were feeding on. If no bees were seen immediately, each plant species (if a tree or shrub) or distinct flower patch was observed for one minute before moving on to the next patch. Where possible, the caste of the individuals (queen, worker or male) was recorded. Sections between flower beds were devoid of flowers and were not surveyed. Although the availability for floral resources was not quantified during each visit, bee forage plants were available throughout the entire survey period.

## Results

### Nectar Foraging Rates

**Figure 3:**
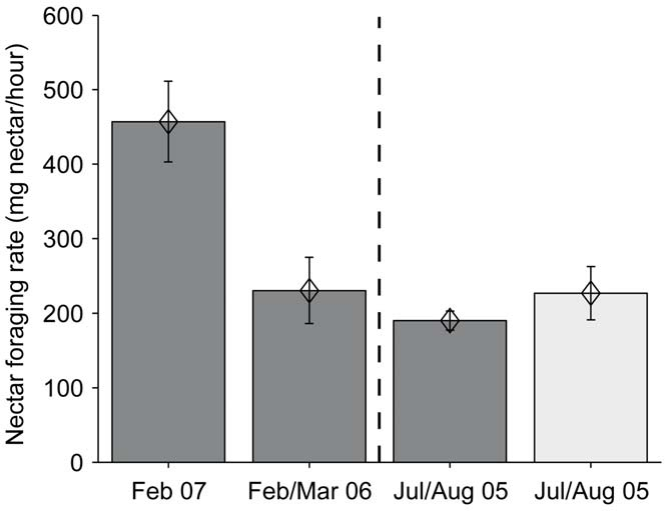
Nectar foraging performance of *B. t. dalmatinus* colonies during different times of year in southern England. The first two bars show the mean (±1 SEM) nectar foraging rates (NFR: mg nectar h^−1^) for the winter observations carried out during this study. The two bars after the dashed line show results from previous observations carried out in the summer, described in [25] and [17], respectively. Observations indicated by dark grey bars were carried out at the same location (Queen Mary University of London, UK) the last observation (light grey) was conducted near Egham, Surrey, UK. Note that the first two bars represent means of workers from one colony each (14 and 10 workers, respectively, whereas the last two bars represent colony means (12 and 5 colonies, respectively).

During the winter foraging observations in 2005/2006 (colony A), 10 foragers were observed performing a total of 75 nectar foraging bouts (mean 7.5±1.0 SEM bouts per bee). During this period, bees successfully foraged (mean nectar foraging rate of 230.5±44.1 mg h^−1^, Fig. 3) at temperatures as low as 3°C. Foraging bouts lasted on average 13.9±1.7 minutes and the bees collected a mean of 40.3±4.6 mg nectar per foraging bout.

In the winter of 2006/2007, 14 foragers from colony B were recorded performing a total of 261 nectar foraging bouts (mean 18.6±2.9 SEM bouts per bee). A mean of 100.3±7.5 mg of nectar were collected per foraging bout, which took on average 21.5±1.9 minutes. This resulted in a mean nectar foraging rate of 457.1±54.3 mg h^−1^ (Fig. 3). This is substantially above the range that has been recorded for *B. t. dalmatinus* in previous experiments at the same location and other locations in southern England in spring/summer (Fig. 3; 12 colonies at Queen Mary with NFRs ranging from 87±8 to 257±18 mg h^−1^, see [25] for details; five colonies tested near Egham, Surrey (suburban/rural) with NFRs ranging from 146.5±27.9 to 440.0±51.3, see [17] for details).

### Pollen Foraging Rates

A total of 97 pollen foraging bouts performed by 14 foragers (6.9±0.9 bouts per bee) were recorded for colony C. The mean pollen intake per bout was 20.7±2.7 mg pollen. Mean bout durations were similar to the ones recorded during the nectar foraging observations (27.9±2.1 min). This resulted in a mean pollen foraging rate of 41.7±5.0 mg pollen h^−1^, which is higher than previous findings during spring (May 2005) at the same location (1 colony with a mean PFR of 15.1±2.6 mg pollen h^−1^; Stelzer, R.J. unpublished data).

### Transect Walks

**Figure 4:**
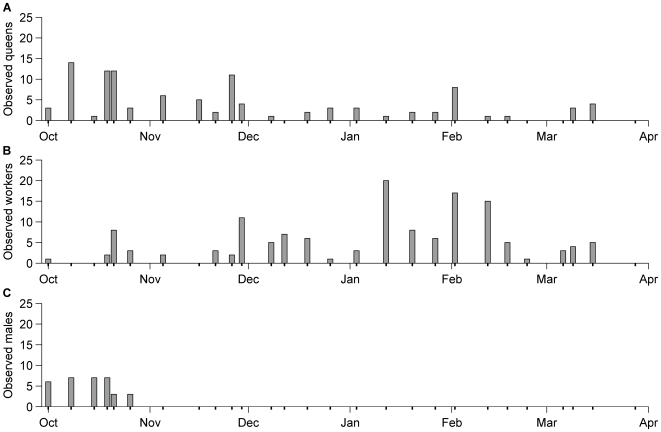
*Bombus terrestris* activity during the winter at Kew Gardens (London, UK). Grey bars represent the number of *B. terrestris* queens (A), workers (B) and males (C) observed during the 27 transect walks conducted during the winter of 2007/2008. Individual transect dates are indicated by bold tick marks on the horizontal axis: six in October, five in November and four in each of the other months.

Both workers and queens of *B. terrestris* could be seen throughout the winter during our transect walks, with up to over 20 individuals recorded during walks in January and February (Fig. 4). At the beginning of our observations in October/November we found a peak in the number of queens (Fig. 4a). Numbers of workers started rising in early December, reaching their maximum in January, before declining again in late February/early March (Fig. 4b), just before the number of observed queens rose again. Males were only seen until early November (Fig. 4c).

**Figure 5:**
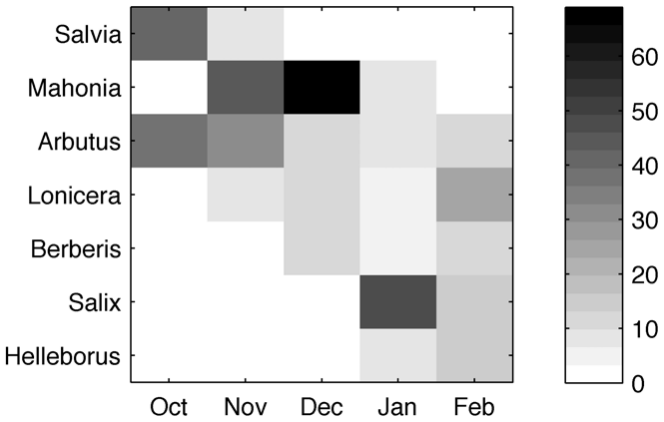
Winter flowering plants frequently visited by *B. terrestris* at Kew Gardens (London, UK). Only the seven genera most frequently visited during the winter of 2007/2008 are shown, see results for species. Colours indicate the proportion of bees seen feeding on these flowers in relation to all observed flower visits during that month.

Only cultivated plants were in flower and the main plants visited by *B. terrestris* were *Arbutus unedo* and *Salvia uliginosa* in October (38.2% and 42.3% of the total recordings during that month, respectively), *Arbutus* spp. (*A. unedo* and *A. x andrachnoides*) (31.2%) and *Mahonia* spp. (*Mahonia x media* ‘Winter Sun’, *Mahonia x media* ‘Charity’ and *Mahonia lomariifolia*) (43.7%) in November, *Mahonia* spp. (69.0%) in December, *Salix aegyptiaca* in January (48.2%) and *Lonicera fragrantissima* (24.1%) in February (Fig. 5). There were also a number of conspicuous, and sometimes abundant, winter flowering plants that were not visited by bumblebees during the survey period. The most notable of these were shrubs such as *Viburnum farreri* (and its hybrids), winter bedding plants (*Viola* spp. and *Cheiranthus* spp.), and autmn crocusses (e.g. *Colchicum autumnale*).

## Discussion

**Figure 6:**
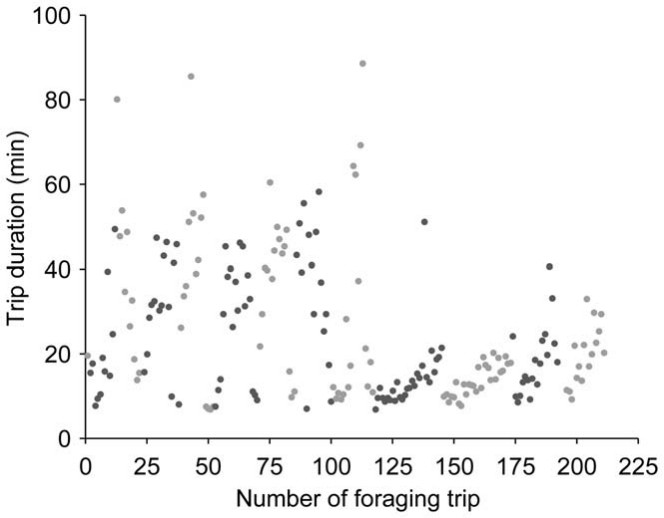
Trip durations for all foraging flights made by one one individual *B. terrestris* worker during January/February 2007. Flights shorter than 5 minutes are not shown. Trips performed during the same day are shown in the same colour. Colours are alternated daily (i.e. day 1 =  light grey, day 2 =  dark grey, day 3 =  light grey and so on).

Our data show that urban areas can represent a rich winter foraging resource for *B. terrestris* and other pollinators. By utilising cultivated winter-flowering plants, especially *Mahonia* spp., high nectar and pollen foraging rates can be achieved, which promote colony fitness [23]. In fact, the foraging rates recorded towards the end of winter were in the top of the range of values recorded in other studies during summer (Fig. 3). The consistently high nectar and pollen foraging rates observed during the late winter experiments clearly resulted from bees being able to collect large quantities of nectar and pollen quickly. The most likely explanation for this is that bees were able to exploit rich food sources close to their nest location. The results of our transect walks show that large multiflorous plants, such as *Mahonia* (*M. x media* and *M. japonica*), are highly visited by *B. terrestris* during winter (Fig. 5). Whilst nectar concentrations of both types of *Mahonia* (33–36% sugar (w/w), see [7]) are well within the range of flowers typically visited by bees (usually 20 to 60% sugar (w/w)), nectar standing crop volumes (4–5 µl) are much greater than typical bee visited flowers (usually <1 µl; [26]). This would potentially allow bumblebee foragers to perform very short foraging trips. Indeed there is evidence that bees find and exploit abundant resources near the nest: figure 6 shows the complete foraging career of one (typical) forager of colony A, automatically recorded by the RFID system over the whole experimental period (i.e. not only during the nectar foraging measurements). After about 115 foraging trips (9 days) the trip durations drop drastically, indicating that the worker might have found a rich food source close by to which it kept returning for the last four days of the experiment. Such rich food sources might not have been available in close proximity to the nest in other observations during summer, or the available ones might have been less profitable due to competition by other pollinator species. During winter, competition should be significantly lower than during spring and summer as few, if any, pollinators are active. This is especially true for bees, although honeybees (*Apis mellifera*) can be seen occasionally on very warm days, but typically not at temperatures below 10°C [27]. Thus, *B. terrestris* would need to visit fewer flowers per trip to collect sufficient pollen and nectar as it would encounter previously unvisited flowers more frequently than bees in the summer. Furthermore, many of the winter flowering plants are large mass flowering shrubs, e.g. *Mahonia*, meaning bees would need to make fewer inter-plant and shorter inter-flower flights in order to visit sufficiently many flowers to fill their crops with nectar or corbiculae with pollen. These combined effects would result in relatively short bout durations in the winter, as observed in this study. However, mean foraging trip durations of about 20 minutes have also been reported during the summer in one study [17], although these bees were also observed to be utilising mass flowering plants (e.g. *Rhododendron* spp. and *Rubus* spp.) within the vicinity of their nests (Ings, T.C., unpublished data).

Our data in this study are based on a limited number of colonies, but even when allowing for intercolony variability in foraging performance [17], [25], [28], the data demonstrate that bees can attain foraging rates at least equal to those during the summer. As a matter of fact, nectar foraging performance of colony B in this study was over 1.7 times greater than the best colony from Raine & Chittka's summer study on 12 colonies in 2008 [25], carried out at the same location in London and using the same commercially available population. Whilst it could be argued that commercial bumblebee colonies are not necessarily representative of native British *B. terrestris* (i.e. they are normally larger than comparable wild colonies), the purpose of our study, at this stage, was to assess the availability of winter forage in urban areas rather than the success of winter active colonies. This was best achieved by using bees from strong colonies kept under optimal conditions (i.e. commercial bees placed in a greenhouse). Our results from two years show that rich foraging resources, exemplified by high foraging rates, are available to bumblebees in urban areas during late winter. The question of whether native bumblebees are able to effectively exploit these resources over the whole winter duration, i.e. establishing a successful second generation in autumn/winter, is initially addressed by our survey data and will be tested in more detail in future experiments.

The data from our detailed winter surveys of a fixed population of bumblebees (Kew Gardens, London) provide good evidence to support the notion that native British *B. terrestris* is establishing a full winter generation in urban areas in southern England [3]–[5]. Caution is necessary to avoid over-interpretation of the changes in queen and worker abundance during the survey period as these may have been influenced by fluctuating weather conditions and food resources. However, the observations of nest founding queens (many were collecting pollen, which is fed to larvae) in October/November, followed several weeks later by workers (Fig. 4a,b), mirrors the pattern observed for a typical spring/summer generation [29]. Whilst this indicates successful establishment of winter colonies, it is difficult to say whether they produced new queens and males. ‘Pristine’, thus potentially new, queens have been observed elsewhere (e.g. Windor Great Park, Surrey and Nailsea, North Somerset) during late winter/early spring (Ings, T.C., personal observation). Thus, the small peak in queen abundance during February/March might be indicative of colonies producing new queens, although they could have been queens emerging from hibernation. The lack of males (which can also be produced by workers if the queen dies) after October suggests that the winter generation in our study population may have failed to produce sexuals. Clearly, further studies on colony maintenance and reproductive success during winter are required.

Although our data gathered from a small number of colonies during the end of the winter represent only preliminary findings, they show that exotic garden plants in warm urban winter climates clearly provide a niche for pollinators, which now seems to be exploited by *B. terrestris*. Observations of winter active *B. terrestris* throughout southern Britain (Fig. 1) show that this phenomenon is not restricted to London, although bees appear to be dependent upon cultivated plants in the absence of native winter flowering plants. It is possible that other pollinator species will follow suit, which in combination with milder winters might relax selection pressure on native plants to flower in the spring/summer period, precipitating further changes in the phenology of pollination systems. Further studies over the whole winter period are necessary to examine this change in pollinator phenology in more detail and to reveal if this change is a response of native bumblebees to climate change or if a possible hybridisation with commercially imported *B. t. dalmatinus*, which naturally have an autumn/winter generation [23], [30], [31], also plays a role.
